# QTc Interval Dispersion in Pediatric Epilepsy: A Case–Control Study From Iran

**DOI:** 10.1111/anec.70094

**Published:** 2025-05-20

**Authors:** Ahmad Talebian, Ali Mohammad Shakiba, Adele Malekipoor, Fatemeh Hafezipour, Mohammad Mahdi Heidari, Fatemeh Talebian, Hamid Reza Gilasi

**Affiliations:** ^1^ Department of Pediatrics Kashan University of Medical Sciences Kashan Iran; ^2^ Clinical Research Development Unit Shahid Beheshti Hospital, Kashan University of Medical Sciences Kashan Iran; ^3^ Department of Pediatrics Faculty of Medicine, Iran University of Medical Sciences Tehran Iran; ^4^ Department of Epidemiology & Biostatistics Kashan University of Medical Sciences Kashan Iran

**Keywords:** child, epilepsy, Kashan, QTc dispersion

## Abstract

**Introduction:**

QT interval corrected dispersion (QTcd) reflects the heterogeneity of ventricular repolarization and has been proposed as a marker for arrhythmic risk in various neurologic and cardiac conditions. The aim of this study was to evaluate QTcd differences between children with epilepsy and healthy controls, with attention to age and antiepileptic drug use.

**Methods:**

A case–control study was conducted on 50 children with epilepsy and 50 age‐ and sex‐matched control children admitted to Shahid Beheshti Hospital in Kashan in 2019. QTcd was manually measured from 12‐lead electrocardiograms (ECGs). Data were analyzed using SPSS version 22.

**Results:**

No overall significant difference was observed in QTcd between groups (*p* > 0.05). However, children with epilepsy under 5 years of age had significantly higher QTcd than controls of the same age (*p* = 0.014). Moreover, QTcd was significantly lower in children with epilepsy receiving medication compared with those not on treatment (*p* = 0.026).

**Conclusion:**

Although overall QTcd did not differ significantly between epileptic and control children, age under five and antiepileptic drug use significantly influenced QTcd. These findings suggest the importance of cardiac evaluation and early treatment in younger patients with epilepsy.

## Graphical abstract

This medical illustration emphasizes QT interval changes in an electrocardiogram (ECG), a crucial factor in children with epilepsy. The magnified QT segment signifies its importance in cardiac monitoring, while subtle neurological elements symbolize the connection between epilepsy and cardiac function.

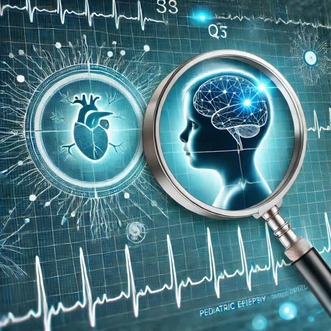

## 1 Introduction

Epilepsy is characterized by recurrent, unprovoked seizures (Fisher et al. 2014). The disorders affect all ages, particularly youngsters, and have social, behavioral, health, and economic effects on patients and families. Epilepsy affects approximately 50 million individuals globally (GCa et al. 2005). Some people have substantial psychological and intellectual disabilities. Epilepsy causes 0.3% of global fatalities, according to the World Health Organization (WHO) (Mbizvo et al. 2019). The central nervous system (CNS) controls cardiac and vascular stimulatory nerve activity (Paton and Spyer 2013; Barman 2020; Herring et al. 2019). Cardiovascular and other organ dysfunction sometimes result from the CNS diseases (Chen et al. 2017). Cardiac symptoms include heart rate fluctuations and ECGs (Zijlmans et al. 2002). In acute neurological illnesses, sympathetic nerve activity increases catecholamine circulation, causing cardiac issues, such as irregular heart rhythms, electrocardiogram (electrocardiogram) abnormalities, ischemic heart damage, and a bad prognosis (Zijlmans et al. 2002; Afsar et al. 2003).

QT dispersion (QTd) is especially impacted by cardiovascular disease (Bazoukis et al. 2020). The heterogeneity of the heart's depolarization may be gauged by observing this event (Prenner et al. 2016). QTd varies depending on the specific neural circuits involved and the location of the brain lesion. QTd is a risk factor marker in a wide range of medical settings, both cardiac and non‐cardiac (Rodríguez‐Jiménez et al. 2019; Hamaguchi et al. 2015; Movahedian et al. 2018). It has been studied in several conditions. Some investigations found that patients had larger QTd than healthy controls (Lazar et al. 2008; Rahar et al. 2016; Akdag et al. 2015; Akbal et al. 2014). QTd increased following admission in acute neurological event patients, according to Lazar et al. It is significantly related to in‐hospital mortality and discharge functional prognosis (Lazar et al. 2008). Sadrnia et al. (2013) found that patients had considerably greater QTd than controls. These findings imply that arrhythmias may cause primary seizures without a known cause.

In order to further examine the results of previous studies on patients with epilepsy (Sheng and Cheng 2017; Koca et al. 2012) as well as the need to understand the etiology and prognosis of these patients, the present study compares QT corrected dispersion (QTcd) in children with epilepsy with the control group in Shahid Beheshti Hospital in Kashan in 2019.

## 2 Materials and Methods

This case–control study was conducted in the pediatric ward of Shahid Beheshti Hospital in Kashan in 2019. All children with and without epilepsy who met the inclusion criteria were evaluated. The study received ethics approval from the Ethics Committee of the Kashan University of Medical Sciences IR.KAUMS.REC.1398.038, and all participants gave written informed consent.

### 2.1 Study Design

Initially, a prepared checklist was used for both the patient and control groups, and then a 12‐channel ECG was prepared from one device on the second day of hospitalization, and QTcd in both groups was manually calculated by a pediatric cardiologist.

To evaluate this rate, we first obtained QT interval and RR distance in 12 ECG leads, and then, QTc was calculated in all 12 ECG leads with Bazett's formula: $\frac{\mathrm{QT}}{\sqrt{\mathrm{RR}.}}$

**FIGURE 1:**
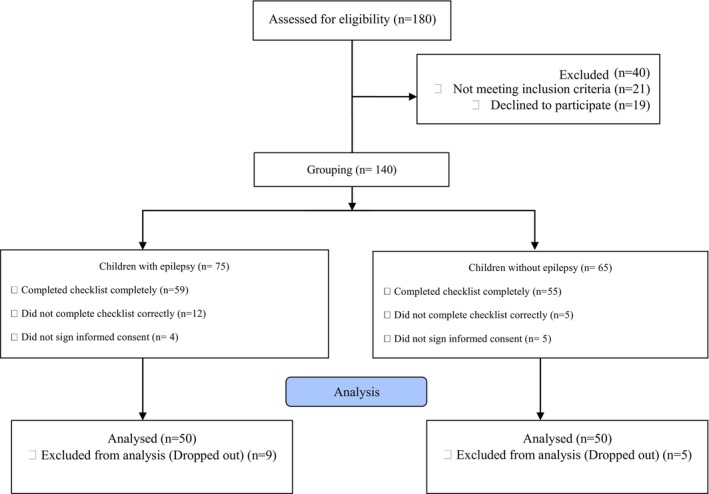
Study flowchart.

Following the previous procedure, we obtained the maximum and minimum number of intervals in milliseconds and we defined cases that had a QTc of 50 milliseconds as a long QTcd. At the same time as taking the ECG, the checklist was asked and recorded by the child's parents (Figure 1).

### 2.2 Inclusion Criteria

#### 2.2.1 In Case Group

Children with epilepsy whose diagnosis was confirmed by a pediatric neurologist based on history and clinical manifestations and also by electroencephalography (EEG).

#### 2.2.2 In Control Group

– Children who do not have a history of infectious, inflammatory, vasculitis, and underlying diseases and have no history of any previous seizures, but with other complaints, have been referred to the pediatric ward of Shahid Beheshti Hospital.
– Absence of progressive brain disease.
– Age 2–12.

### 2.3 Exclusion Criteria

– History of cardiovascular disease (ischemic, valvular, abnormal rhythm, or cardiomyopathy).
– Altered mental state due to metabolic disorders, space‐occupying lesion, and infection.
– History of using the known drug affecting the repolarization parameters in the ECG, including digoxin, antidepressants, phenothiazines, tricyclic antidepressants, lithium carbonate, erythromycin, and levodopa.
– Patients with ECG show branching and sinus block.

### 2.4 Data Analysis

SPSS version 22 (SPSS Inc., Chicago, IL, USA) was used for statistical analysis of the data. The chi‐squared test was used to compare qualitative features between groups. To ensure that all quantitative parameters were normally distributed, the Kolmogorov–Smirnov test was run. The Mann–Whitney *U*‐test was used for non‐normally distributed data, whereas the Student *t* test was employed for normally distributed variables. The significance threshold was set at a *p* value of less than 0.05.

## 3 Results

A total of 100 people (50 children with epilepsy and 50 control children) participated in this study. Table 1 shows the frequency distribution of children by sex and age.

**TABLE 1:** Distribution of children in two groups according to gender and age.

Variable	Group				*p*
	Epilepsy		Control		
	Frequency	Percentage	Frequency	Percentage	
Gender					
Boy	27	51.9	25	48.1	0.689
Girl	23	47.9	25	52.1	
Age					
Less than 5 years	21	52.5	19	47.5	0.683
5–10 years	29	48.3	31	51.7	

Table 1 shows that age and sex variables have no significant relationship with the studied groups (*p* > 0.05). Therefore, it can be concluded that the studied groups are homogeneous in terms of age and sex. In other words, age and sex were not confounding variables (Table 2).

**TABLE 2:** Comparison of QTc dispersion values between the two groups.

Variable	Group		*p*
	Epilepsy	Control	
**QTcd**			
msec	41.72 ± 6.12	39.98 ± 5.64	0.142
Normal (less than 50)	44 (48.9)	46 (51.1)	0.505
Long (50 and up)	6 (60)	4 (40)	

There is no significant difference between QTcd values, both in terms of amount of milliseconds and in terms of normal/long, between the epileptic and control groups (*p* > 0.05). In other words, no significant relationship was observed between QTcd and epilepsy (Table 2).

The results of Table 3 show that there is no significant difference in QTcd values between the two groups by sex (*p* = 0.950), but there was a significant difference in terms of age (*p* = 0.014); in the age sub group of less than 5‐year‐olds, the amount of QTcd in the epileptic group (43.86 ± 5.90) is significantly higher than the control (38.63 ± 5.17).

**TABLE 3:** Comparison of QTc dispersion values (milliseconds) between the two groups by sex and age.

Variable	Gr	Oup	*p*
	Epilepsy	Control	
Gender			
Boy	42.22 ± 6.09	40.60 ± 6.31	0.950
Girl	41.13 ± 6.23	39.36 ± 4.91	
Age			
5 years >	43.86 ± 5.90	38.63 ± 5.17	0.014
5–10 years	40.17 ± 5.90	40.80 ± 5.83	

QTcd was significantly lower in children with epilepsy receiving antiepileptic medication (39.06 **±** 4.98) than in children who did not take the drug (43.09 **±** 6.27). Also, no significant difference was observed in the amount of QTcd in children with epilepsy who had seizures up to two times compared with those who had seizures more than two times (*p* = 0.424) (Table 4).

**TABLE 4:** QTc dispersion values (milliseconds) in the epileptic group according to drug use and number of seizures.

Variable	QTcd	*p*
	Mean ± SD	
Taking medication		
Positive (17 Normal)	39.06 **±** 4.98	0.026
Negative (27 normal and 6 long cases	43.09 **±** 6.27	
Number of seizures		
< 2 times (19 normal and 4 long cases)	42.48 **±** 5.53	0.424
≥ 2 times (25 normal and 2 long cases)	41.07 **±** 6.61	

## 4 Discussion

In this study, QTcd in children with epilepsy with a control group in Shahid Beheshti Hospital in Kashan in 2019 was compared. A total of 100 children were studied, of which 50 were control children and 50 were epileptic. Six children in the epilepsy group and four children in the control group had prolonged QTcd. The results of the present study also showed that the rate of QTcd in milliseconds in children with epilepsy was not significantly different from that of the control group and this lack of difference in terms of gender continued, but in terms of age, a significant difference was observed in the age group less than 5 years and the amount of QTcd in the epileptic group is significantly higher than that of the control group. It was also found that QTcd, which was studied in the patients with epilepsy, did not differ significantly in the number of seizures and the underlying disease. However, in terms of medication, children with epilepsy who did not take medication had higher QTcd than those of children with epilepsy taking medication.

Increased QTcd interval distribution can be seen in patients with various heart diseases, such as myocardial infarction, left ventricular hypertrophy, long QT syndrome, heart failure, and aortic stenosis. In addition to the aforementioned diseases, increased distribution of QTcd interval in non‐cardiac diseases has also been studied (Panikkath and Panikkath 2015). One of the disorders that have been previously compared with healthy control groups in terms of QTd rate is epilepsy (de Sousa et al. 2017). In case–control study of Sadrania et al., which was conducted in 2013 in Arak on children with primary seizures without a specific cause and also children without seizures, it was found that QTd in the group of patients was significantly higher than in the control group. However, syncope and sudden death were not significantly different between the two groups. These results suggest that primary seizures without a specific origin may be due to an arrhythmia (Sadrnia et al. 2013). Akbal et al. (2014) said that QTd in the group of children with epilepsy was higher than in the control group, which is in contradiction with the present findings. There was also no significant difference in the rate of QTd between children undergoing antiepileptic treatment and the group of patients without treatment. However, in the present study, the amount of QTcd was significantly lower among children who took the drug than among children who did not.

In a study conducted by Movahedian et al. (2016) at Kashan University of Medical Sciences on the distribution of QTcd in children with stroke attacks (seizure mimics), the values obtained are inconsistent with the values obtained in our study and indicate a higher mean and standard deviation. QTcd was higher in children with string attacks than in controls. In justifying the differences between the present results and the mentioned study, we can also point out the differences between the studied diseases and their different effects on QTc values. Therefore, it seems that conducting such studies in each region requires the use of a matched control group to compare the values in the patient group with normal values in accordance with the genetic characteristics of the same region.

Besides comparing QTcd between epileptic and healthy children, factors like seizure frequency and medication use were also assessed. In the present study, no significant difference was observed in QTcd in children with epilepsy who had seizures up to two times compared with those who had more than two seizures.

Akın et al. (2018) found that if in children with breath‐holding spells QT and QTc did not show an increase, the increase in QTd could not be attributed to autonomic dysfunction.

In the study, Bornaun et al. (2017) found that 33 children did not take the drug, 32 children did not have long QT Dispersion, and only one case had long QTd. One child had prolonged QTd in addition to taking sodium valproate, but nine children taking sodium valproate did not have prolonged QTd. In the present study, all children who took the drug had normal QTc Dispersion and, among those who did not take the drug, six had long QTcd. This finding in the present study showed a significant difference between drug users and non‐drug users, indicating that medication use was associated with lower QTcd. Drugs are likely to be effective in causing these changes and why this finding can indicate the pharmacological effect of the drug in preventing these ECG changes.

When comparing results from research that employ quantitative criteria, one common source of disagreement is the use of different measuring methods and/or calculation standards. Changes between the results of the current research and those of certain other studies may be due, in part, to changes in the technique of measuring QTd in children, in addition to genetic, ethnic, and regional factors.

QT dispersion has been extensively studied internationally. In contrast to the current investigation, Yi et al. employed just 11 leads to quantify the QT distribution, and they left out the V1 lead as its T‐wave is often smooth and unmeasurable. Lead III is the second possible indication of trouble. It is recommended that any additional leads with insufficient T‐wave amplitudes be left out of the analysis (Yılmaz et al. 2018). This approach results in fewer QTd identifications and inconsistencies between the current research and its predecessors. It is possible that the QTc distance distribution calculated from just three of the typical ECG leads is just as useful as the one calculated from all of the leads.

The advantage of standard measuring is the simplicity of execution and the omission of problems associated with lead modulation (Magrì et al. 2017). The main problem in detecting the end of the T‐wave is the presence of the U‐wave. The change in QRS onset in a 12‐lead ECG can be up to 24 msec. In manual measurements, increasing the paper speed is not useful to reduce the measurement error. Therefore, a speed of 50 mm/s was suggested for ECG recording (Murray et al. 1994).

The above facts demonstrated that the source of error in the difference of results should not be forgotten. An interobserver variability of 25%–40% has been reported for QT dispersion measurements. Today, the gold standard is manual measurement using a digital screen. Calipers The lines used in manual measurements play the most important potential role in measuring error and difference in results. The available automated algorithms are unfortunately no better than human‐measured ones. A relatively small error in QT measurement exacerbates the error in QT distribution. An additional problem is the periodic changes in the QT interval (Akın et al. 2018).

Due to the methodological problem associated with measuring QT distribution, the normal values of QT distribution are not well defined. The general consensus is that values between 30 and 60 ms are considered normal. Due to the large differences between the observations of different individuals, it is suggested that the researcher himself obtains normal values based on a study on the ECG of healthy individuals. In the present study, we set the QTd to 50 milliseconds (Bazett 1997). Although QT distribution is not a replacement for more invasive methods of electrophysiological study, it is a simple and helpful parameter for electrophysiological evaluation. However, the diagnostic value of QT distribution is limited due to variations in the measurement of QT distribution by different individuals as well as variations in the standardization of QT distribution in children (Lederman et al. 2019).

Limitations of our study include the relatively small sample size, manual QTcd measurement by a single observer, and lack of interobserver reliability assessment. These factors may affect generalizability and accuracy. Future studies with larger sample sizes and automated ECG analysis are recommended to validate and expand on these findings.

## 5 Conclusion

Although QTcd did not show a universal difference in children with epilepsy compared with controls, specific subgroups, such as younger children or those not on medication showed significant variations. Incorporating routine QTcd assessment, particularly in young or untreated patients with epilepsy, may provide insights into arrhythmogenic risks and guide clinical management. According to research, children with epilepsy have a long QT distribution and are prone to arrhythmia; hence, cardiac examination for arrhythmia and long QT syndrome should be included in epileptic diagnosis. Future studies should aim to replicate these findings.

## Author Contributions

A.T., A.M.S., and A.M. designed the project; F.H. performed the study; H.R.G. analyzed data; and M.M.H. and F.T. wrote the article.

## Consent

The informed consent was obtained from the parents to enter the study at the beginning of the study.

## Conflicts of Interest

The authors declare no conflicts of interest.

## Data Availability

Research data are not shared.
